# UK practice on incidentally detected non-functioning pituitary microadenomas: analysis of two national surveys during a 12-year interval

**DOI:** 10.1007/s11102-022-01290-4

**Published:** 2022-11-25

**Authors:** Ross Hamblin, Athanasios Fountas, Miles Levy, Niki Karavitaki

**Affiliations:** 1 Institute of Metabolism and Systems Research, College of Medical and Dental Sciences, University of Birmingham, IBR Tower, Level 2, Birmingham, B15 2TT UK; 2 Centre for Endocrinology, Diabetes and Metabolism, Birmingham Health Partners, Birmingham, UK; 3 Department of Endocrinology, Queen Elizabeth Hospital, University Hospitals Birmingham NHS Foundation Trust, Birmingham, UK; 4 Department of Endocrinology, University Hospitals of Leicester NHS Trust, Leicester, UK

**Keywords:** Pituitary, Non-functioning, Microadenoma, Incidentaloma, Pituitary tumour

## Abstract

**Purpose:**

The optimal management approach for presumed non-functioning pituitary microadenomas (microNFPAs) remains unclear. Our aim was to capture current UK practice and identify changes with time.

**Methods:**

Two online surveys investigating clinicians’ approaches were performed in 2009–2010 and 2021–2022 (advertised through Society for Endocrinology UK).

**Results:**

150 and 214 clinicians participated in the 2021 and 2009 survey, respectively (response rates 31.2% and 35.4%, respectively). At baseline, 2021 survey respondents were more likely to measure IGF-1 (96.0% vs 74.1%, *p* < 0.001) and morning cortisol (87.9% vs 62.6%, *p* < 0.001), and less likely GH (26.2% vs 42.6% *p* = 0.002), 24 h urine free cortisol (3.4% vs 23.2%, *p* < 0.0001) or dynamically assess adrenal reserve (11.4% vs 30.4%, *p* < 0.001). 47.2% of clinicians in 2021 would reassess pituitary function annually until discharge (in absence of tumour growth/symptoms). The 2021 survey respondents were more likely to stop imaging at or before 3 years (81.7% vs 44.3%, *p* < 0.001) and at or before 5 years (86.6.% vs 72.9%, *p* = 0.002), whilst 2009 survey respondents were more likely to continue imaging beyond 5 years (24% vs 7%, *p* < 0.001). Responses on imaging frequency/intervals showed notable variability in both surveys.

**Conclusions:**

Diagnostic and management approaches for microNFPAs have evolved in the UK. Biochemical investigations are performed in accord with consensus guidelines, though many clinicians perform annual biochemical surveillance without tumour growth/symptoms. A small number of clinicians request imaging beyond 5 years, but the frequency of imaging intervals until discharge remains variable. Robust evidence on the long-term natural history of microNFPAs is necessary to unify clinician approach.

**Supplementary Information:**

The online version contains supplementary material available at 10.1007/s11102-022-01290-4.

## Introduction

Non-functioning pituitary adenomas (NFPAs) are benign tumours with a reported prevalence of 7-41.3/100,000 population [1, 2]. They do not cause clinical manifestations associated with hormonal hypersecretion and they become clinically apparent when they are large enough to exert pressure effects to surrounding structures. Smaller NFPAs (usually microadenomas) may remain undiagnosed during the life span or are incidentally detected on imaging performed for unrelated reasons [3]. In earlier series, high prevalence of incidental pituitary tumours or “pituitary incidentalomas” had been suggested; 10–38% of healthy volunteers (unknown to have pituitary disease) had been shown to have pituitary abnormalities on magnetic resonance imaging (MRI) [4, 5]. Estimates from combined autopsy studies with an amounted sample size of 18,902 subjects reported incidentally detected pituitary adenomas in 10.7% of them, with all but seven being microadenomas [3]. Furthermore, in a large population study from Northern Finland covering the period between 1992 and 2007, 51% of the total NFPAs (18% of which were microadenomas) were detected incidentally; in this report, a significant rise in the standardized incidence rates of incidentalomas was confirmed over the years (0.59 vs 1.6 per 100,000 from 1992 to 1999 to 2000 to 2007) [6]. Data from a US population study also suggest a three-fold increase in the number of pituitary incidentalomas detected between 2004 and 2018 [7].

The natural history of incidental microNFPAs is still not clearly established; whilst the autopsy findings suggest that most go unnoticed and progression from micro- to macroadenoma is a rare event, rates of tumour growth and hypopituitarism vary considerably within the literature (between 0 and 53% [8–19] and 0 and 50% [8–19], respectively). Such breadth in reported outcomes relates with heterogeneity in the methodology and design of the studies, differences in the diagnostic approach for confirming hypopituitarism, duration of follow-up, as well as inclusion of incidentally found lesions other than NFPA in the analyses (e.g., functioning adenomas or cystic pathologies [9, 17, 19, 20]). Furthermore, evidence to date arises mostly from reports with a limited duration of radiological follow-up [16].

A survey amongst UK and US endocrinologists exploring their approach in the evaluation of incidental pituitary adenomas was conducted in 1997 [21] and demonstrated large variation in clinical practice. Since then, the publication of guidelines on the diagnostic and management approach for such tumours has, therefore, been welcome, though caution may be needed for some recommendations that rely solely on expert opinion [22–24].

Given the rising number of incidentally detected microNFPAs, their potential implications (for both patients and the health care system) and the on-going uncertainties on their long-term management, two UK-wide surveys were released in 2009–2010 and 2021–2022. The primary aim of our study was to capture the contemporary practice of UK clinicians in the investigation and management of incidental (presumed) microNFPAs and the secondary objective was to identify changes in clinical practice since the 2009–2010 survey.

## Methods

Two online surveys on the diagnostic and management approach for a presumed microNFPA were endorsed by the Society for Endocrinology (SFE) UK and were released with a 12-year difference (2009 and 2021).

The 2009 survey included a short clinical case scenario of a 25-year-old female discovered to have a 5 mm pituitary microadenoma on MRI after investigations for chronic non-specific headaches; the patient had normal physical examination, regular menses, and absence of galactorrhoea. A series of questions then followed asking respondents how likely they were to adopt a particular investigation or management approach (‘1’ representing ‘never’ to ‘4’ representing ‘always’); option for free text views was also included for two questions (Suppl Fig. 1). An online link to the survey was disseminated to the members of the SFE via e-mail.

The 2021 survey included a questionnaire with eight questions, each of which gave a list of investigations and management options the respondent would choose when faced with a patient with an incidentally discovered (presumed) microadenoma. There was no restriction on the number of investigations selected, and additional free-text views could be provided at the respondent’s discretion (Suppl Fig. 2). The survey was advertised by the SFE (on the website, in the monthly news bulletin and during the British Endocrine Society Annual meeting 2021) and the members of the SFE Neuroendocrine Network were invited to participate by e-mail. The survey remained open for 12 months. The participants returned the questionnaires online or on paper.

Responses were analysed for the whole group and for subgroups of the participants in each survey. Comparisons of responses in the two surveys were also performed; to allow comparison between them, responses ‘often’ or ‘always’ in the 2009 survey were considered as positive for each specific question.

### Statistical analyses

Numbers and percentages were used for categorical variables. Chi-square test was used to compare differences between categorical variables. Where expected values were less than 5 in more than 20% of cells, Fisher’s exact test was applied. *p* values < 0.05 were deemed significant. Statistical Analyses were conducted with IBM SPSS statistics for Mac, Version 28 (IBM Corp., Armonk, NY, US).

## Results

### Respondent characteristics

In total, 150 clinicians participated in the 2021 survey (response rate 150/481, 31.2–69% at consultant grade) and 214 in the 2009 survey (response rate 214/604, 35.4–77% at consultant grade). Their characteristics are shown in Table 1.

**Table 1:** Characteristics of responders in surveys of 2009 and of 2021

	2009 Survey (n = 214)	2021 Survey (n = 150) ^*†*^
Clinician grade Number (percentage)		
Consultant	165 (77.1%)	104 (69.3%) *
Specialist registrar (trainee)	49 (22.9%)	44 (29.3%)
Other	N/A	2 (1.3%) **
Type of practice Number (percentage) ***		
Secondary care hospital	80 (43.2%)	53 (35.3%)
Tertiary care hospital	105 (56.8%)	96 (64.0%)
Private practice	0 (0%)	1 (0.7%)

*N/A* not applicable

^†^Responses from the 2021 survey were from England (South-West, South-East, North-West, North-East, West Midlands, East Midlands, East of England and Yorkshire and Humber), Wales, Scotland, Northern Ireland, and the Isle of Man

*95.3% endocrinologists, 4.7% neurosurgeons

**1 Endocrine Nurse Consultant and 1 Physician Associate

***Total number of respondents in 2009 survey: 185

### Biochemical screening at baseline

#### 2021 Survey

All respondents to the questions on investigations at first review indicated they would perform pituitary function tests in the initial evaluation of an incidentally detected pituitary microadenoma (n = 149). Of those, 97.3% and 96% would measure prolactin and insulin-like growth factor-1 (IGF-1), respectively. Follicle-stimulating hormone (FSH), luteinizing hormone (LH) and gonadal hormones would be checked by 96.6% and thyroid stimulating hormone (TSH) and free thyroxine (fT4) by 95.3% of respondents. Morning cortisol measurement would be requested by 87.9% of the participants and 11.4% would perform a Short Synacthen Test (SST); 7% of clinicians would request both morning cortisol and SST. In comments provided as free text, a further 6% indicated they would perform SST in the event of suboptimal morning cortisol value, or if clinically suspected adrenal insufficiency, or in the case of a large microadenoma. Screening for Cushing’s [overnight dexamethasone suppression test and/or 24 h urine free cortisol (24 h UFC)] would be organised by 12.8% of respondents with a further 11.4% mentioning they would only screen if clinical features were consistent with cortisol excess. Plasma and urine osmolalities would be checked by 6.7%, whereas 2% would only do so in the context of clinical suspicion of water balance abnormalities (Table 2).

**Table 2:** Responses for biochemical investigations that would be requested following the incidental detection of a pituitary microadenoma in the 2009 and 2021 surveys

Biochemical investigation Number of positive responses/number of responders to each question (percentage)	2009 Survey	2021 Survey	*p* value
Prolactin	204/213 (95.8%)	145/149 (97.3%)	0.438
IGF-1	152/205 (74.1%)	143/149 (96.0%)	< 0.001
GH	84/197 (42.6%)	39/149 (26.2%)	0.002
Gonadal function			
FSH/LH and gonadal hormones		144/149 (96.6%)	
FSH/LH	155/204 (76.0%)		
Oestradiol	118/201 (58.7%)		
Thyroid function			
TSH + free T4		142/149 (95.3%)	
TSH	187/209 (89.5%)		
Free T4	193/209 (92.3%)		
Morning cortisol	124/198 (62.6%)	131/149 (87.9%)	< 0.001
SST/dynamic test of ACTH reserve^†^	59/194 (30.4%)	17/149 (11.4%)	< 0.001
		A further 9 would perform dynamic testing if clinical features, suboptimal morning cortisol, or if a large microadenoma	
Screen for hypercortisolaemia			
ONDST		17/149** (11.4%)	##
24 h UFC	44/190 (23.2%)	5/149** (3.4%)	< 0.0001
		A further 17 would screen for hypercortisolaemia if relevant clinical features	
LDDST	41/191 (21.5%)		##
Plasma and Urine osmolalities		10/149 (6.7%) A further 3 commented they would measure depending on clinical suspicion	
Other tests *Data are shown as absolute number of responses*			Statistical comparison not performed due to small numbers
Electrolytes	4	7	
Free T3	1	2	
HbA1c/glucose	1	2	
Bone profile	7	1	
Lipids	1	1	
PSA		1	
Calcitonin and chromogranin	1		
24-h urinary Catecholamines	1		
POMC	1		
Macroprolactin	1		
Chest X-ray	1		
AFP and HCG	1		
Ferritin	1		
Serum ACE	1		

#### 2009 Survey

Prolactin would be checked by 95.8%, fT4 by 92.3% and IGF-1 by 74% of the participants. Measurement of gonadotropins would be requested by 76% and oestradiol by 58.7%. Measurement of morning cortisol would be organised by 62.6%, whereas 30.4% would perform dynamic testing for ACTH reserve. In terms of screening for Cushing’s, 23.2% would request 24 h UFC and 21.5% would arrange a low dose dexamethasone suppression test (Table 2).

### Biochemical follow-up

#### 2021 Survey

Responses on biochemical follow-up were received by 142 participants; 47.2% (n = 67) would reassess pituitary function annually until discharge, whilst 52.1% (n = 74) would only repeat hormonal evaluation in the presence of tumour enlargement or if clinical suspicion of new pituitary dysfunction. One clinician (0.7%) indicated they would reassess at 2–3 years or earlier, if clinical suspicion.

#### 2009 Survey

Biochemical follow-up was not assessed in the 2009 survey.

### Visual assessment

#### 2021 Survey

Amongst 149 responders, 15.4% (n = 23) would perform visual assessment at baseline and a further 6% (n = 9) would request this if the tumour was close to the optic pathways on imaging, or if the tumour had suprasellar extension, or if there were concerns for visual morbidity.

#### 2009 Survey

Forty-seven of 190 responders (24.7%) would perform formal plotting of visual fields at baseline.

### Imaging follow-up and discharge approaches

#### 2021 Survey

Amongst 148 clinicians who responded on this section, 4.1% (n = 6) would discharge the patient after the initial baseline imaging, provided the investigations from the basal review were consistent with a microNFPA. Of these, three would offer advice on discharge [re-referral, if new visual field abnormalities (n = 1), or if symptomatic (n = 1), and one would provide reassurance].

**Fig. 1:**
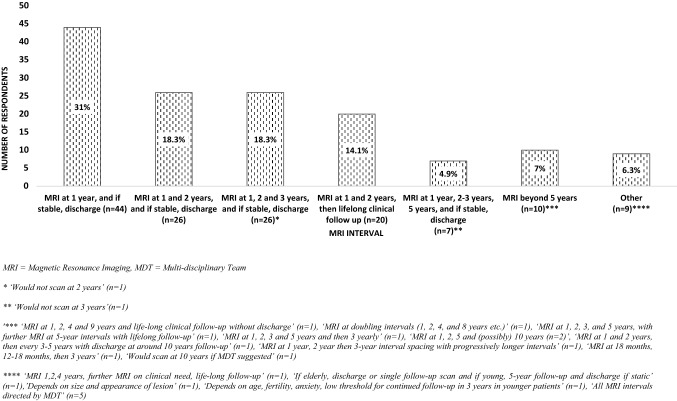
Imaging surveillance intervals chosen by 142 respondents of the 2021 survey. Specific comments provided by the participants are shown below per interval category

Continued imaging surveillance would be offered by 95.9% of the respondents with 81.7% (n = 116) requesting imaging up to three years after the baseline scan (31% at one year, followed by discharge if stable; 18.3% at one and at two years, followed by discharge if stable; 18.3% at one, two and three years followed by discharge if stable, and 14.1% at one and at two years combined with life-long clinical follow-up). A further 4.9% (n = 7) would image for up to five years, whilst 7% (n = 10) of clinicians would continue imaging monitoring beyond 5 years. Further details on the imaging surveillance responses for the 2021 survey are shown in Fig. 1.

#### 2009 Survey

Amongst 208 respondents, 5.3% (n = 11) would not arrange follow-up imaging. Of the remaining 94.7% (n = 197) who would consider radiological surveillance; 3.6% (n = 7), 34.5% (n = 68), 51.8% (n = 102), and 9.6% (n = 19) would request first follow-up imaging at less than 6 months, between 6 and 12 months, at 12 months, or after 12 months, respectively. 0.5% (n = 1) did not specify when they would perform next MRI.

**Fig. 2:**
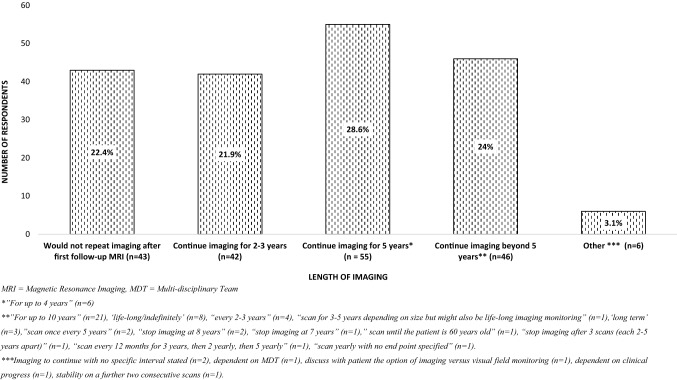
Imaging follow-up reported by 192 respondents of the 2009 survey. Specific comments provided by the participants are show below each category column

Of 192 respondents who provided details on length of radiological surveillance, 22.4% (n = 43) would not perform further imaging after one interval MRI (providing stability), whilst 21.9% (n = 42), 28.6% (n = 55) and 24% (n = 46), would continue imaging up until 2–3 years, 5 years, and beyond 5 years, respectively. Further details on the length of imaging surveillance responses for the 2009 survey are shown in Fig. 2.

### Factors influencing discharge of the patient

#### 2021 Survey

**Fig. 3:**
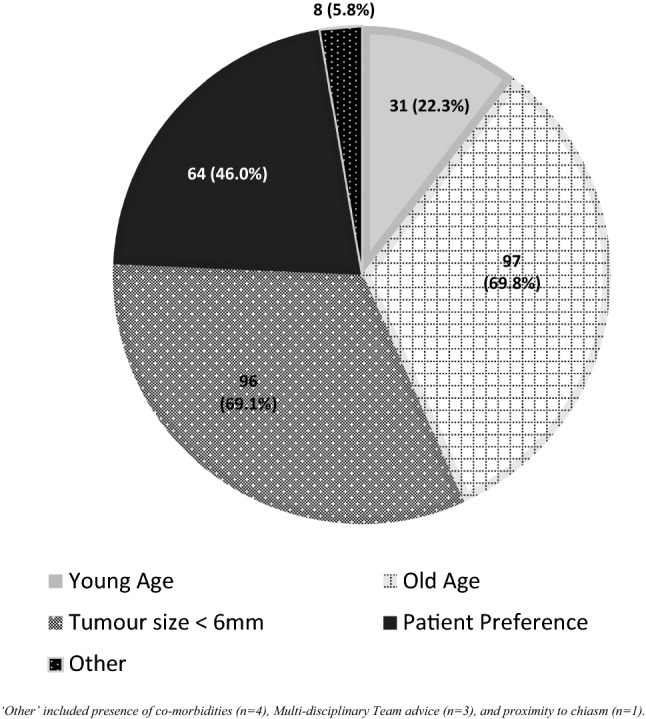
Factors influencing decision on discharge of the patient (n = 139) shown as absolute number of responses and percentage in parentheses. ‘Other’ included presence of co-morbidities (n = 4), Multi-disciplinary Team advice (n = 3), and proximity to chiasm (n = 1)

From the 142 respondents who would continue imaging surveillance, 139 reported factors influencing their decision to discharge the patient. These are highlighted in Fig. 3; most commonly reported were old age (69.8%), tumour size < 6 mm (69.1%) and patient preference (46%).

#### 2009 Survey

Factors influencing patient discharge were not explored in the 2009 survey.

### Comparisons

Comparison of the two surveys showed significant differences in a number of responses (Table 2).

At baseline assessment, the 2021 survey respondents were more likely to measure IGF-1 (96.0% vs 74.1%, *p* < 0.001) and morning cortisol (87.9% vs 62.6%, *p* < 0.001), and less likely to request GH measurement (26.2% vs 42.6% *p* = 0.002), dynamically assess adrenal reserve (11.4% vs 30.4%, *p* < 0.001) or perform 24 h UFC measurements (3.4% vs 23.2%, *p* < 0.0001) or dexamethasone suppression tests (11.4% vs 21.5% *p* = 0.009). Comparison of assessment of the thyrotroph or gonadotroph axes was not possible due to differences in the format of the questions. Fewer respondents in the 2021 survey would arrange visual assessment (15.4% vs 24.7%, *p* = 0.036). There was no difference in the number of respondents who would perform repeat imaging (2021 survey 95.9% vs 2009 survey 94.7%, *p* = 0.590), though 2021 respondents were more likely to stop imaging surveillance at or before 3 years (81.7% vs 44.3%, *p* < 0.001) and at or before 5 years (86.6.% vs 72.9%, *p* = 0.002). Respondents of the 2009 survey were more likely to continue imaging beyond 5 years (24% vs 7%, *p* < 0.001).

In the 2021 survey, clinicians working in secondary care were more likely to perform plasma and urine osmolalities (13.2% vs 3.2%, *p* = 0.035) and imaging at one year, followed by discharge (44.0% *vs* 23.1%, *p* = 0.010). Clinicians working in tertiary care were more likely to perform annual assessment of pituitary function irrespective of tumour stability or absence of new relevant symptoms (54.0% vs 36.0%, *p* = 0.042), (Table 3).

**Table 3:** Comparison of responses given in the 2021 survey between clinicians working in tertiary and secondary care hospitals

	Tertiary care hospitals	Secondary care hospitals	*p* value
Biochemical investigation			
Number of positive responses/number of responders to each question (percentage)			
Prolactin	92/95 (96.8%)	52/53 (98.1%)	1.000
IGF-1	93/95 (97.9%)	50/53 (94.3%)	0.350
GH	30/95 (31.6%)	9/53 (17.0%)	0.053
FSH/LH and gonadal hormones	93/95 (97.9%)	51/53 (96.2%)	0.618
Morning cortisol	84/95 (88.4%)	47/53 (88.7%)	0.962
SST	10/95 (10.5%)	7/53 (13.2%)	0.624
TSH + free T4	91/95 (95.8%)	50/53 (94.3%)	0.701
24 h UFC	3/95 (3.2%)	2/53 (3.8%)	1.000
ONDST	12/95 (12.6%)	5/53 (9.4%)	0.559
Plasma and urine osmolalities	3/95 (3.2%)	7/53 (13.2%)	0.035
Visual assessment	17/95 (17.9%)	6/53 (11.3%)	0.290
Discharge	3/94 (3.2%)	3/53 (5.7%)	0.668
Imaging			
MRI at 1 year and if stable discharge	21/91 (23.1%)	22/50 (44.0%)	0.010
MRI at 1,2 years then discharge	21/91 (23.1%)	5/50 (10.0%)	0.055
MRI at 1,2,3 years then discharge	18/91 (19.8%)	8/50 (16.0%)	0.580
MRI at 1 year, 2/3 years, 5 years then discharge	7/91 (7.7%)	0/50 (0%)	0.051
MRI beyond 5 years	6/91 (6.6%)	4/50 (8.0%)	0.743
MRI at 1, 2 years then lifelong follow-up	11/91 (12.1%)	9/50 (18.0%)	0.336
Annual pituitary function testing	49/91 (54.0%)	18/50 (36.0%)	0.042

*IGF-1* insulin-like growth factor-1, *GH* growth hormone, FSH follicle stimulating hormone, *LH* luteinizing hormone, *TSH* thyroid stimulating hormone, *T4* thyroxine, *SST* Short Synacthen test, *ACTH* adrenocorticotropic hormone, *24 h UFC* 24 h urinary free cortisol, *ONDST* overnight dexamethasone suppression test, *LDDST* low dose dexamethasone suppression test, *T3* triiodothyronine, *PSA* prostate specific antigen, *HbA1c* glycated haemoglobin, *POMC* proopiomelanocortin, *AFP* alpha fetoprotein, *HCG* human chorionic gonadotropin, *ACE* angiotensin converting enzyme

^†^SST only option provided in 2021 survey

**In total, 19 out of 149 would arrange screening for Cushing’s with 3 of them requesting both ONDST and 24 h UFC

^##^p = 0.009 after comparison of those arranging a LDDST (Survey 2009) and an ONDST (survey 2021)

*IGF-1* insulin-like growth factor-1, *FSH* follicle stimulating hormone, *LH* luteinizing hormone, *TSH* thyroid stimulating hormone, *T4* thyroxine, *SST* short synacthen test, *UFC* urinary free cortisol, *ONDST* overnight dexamethasone suppression test, *MDT* multi-disciplinary team, *MRI* magnetic resonance imaging

## Discussion

Our findings from two national UK clinician surveys released 12 years apart suggest that over time, the approach to the investigation and management of microNFPAs has changed in a number of areas. Clinicians in 2021 were more likely to perform IGF-1 and morning cortisol measurement; whilst less likely to request random GH measurement, dynamic testing of adrenal reserve, or 24 h UFC measurement. They were also more likely to stop imaging surveillance by 3 or 5 years but their choice of imaging frequency/interval was variable.

In agreement with international guidelines and expert recommendations, nearly all clinicians in both surveys would request prolactin measurement in the diagnostic work-up of an incidentally found pituitary microadenoma [22–26]. This is justified by the high prevalence of prolactinomas presenting to clinical services, the majority being microadenomas [2, 27]. Notably, in two large population studies, 6–12% of prolactinomas were identified following evaluation of an incidentally detected pituitary mass [6, 28]. Screening is further justified based on cost-analysis, with a single prolactin measurement reported to be most cost-effective investigation in the evaluation of a pituitary microincidentaloma [29]. IGF-1 measurement was selected by nearly all respondents in the 2021 survey and is currently recognised as the initial screening test for acromegaly [22–24, 30]. Acromegaly is attributed to a microadenoma in 14–32% of cases [2, 31, 32]. Smaller tumours are more likely to lead to successful surgical results [33] and screening is, therefore, recommended by relevant guidelines [22–24, 30]. It is of interest that clinicians participating in the 2009 survey would less often check IGF-1 and more frequently random GH measurement, reflecting the evolution in the diagnostic approach [30].

In the 2021 survey, 11.4% of clinicians would perform an overnight dexamethasone suppression test and 3.4% would request 24 h UFC measurement as screening for Cushing’s disease. A further 11.4% would test only in the presence of relevant clinical features. In the 2009 survey, 23.2% of participants would check 24 h UFC and 21.5% would request a low dose dexamethasone suppression test. Investigation for Cushing’s disease, in the absence of a high index of clinical suspicion or pre-test probability, invariably increases the risk of false-positives and potential for unwarranted investigation and anxiety [34, 35]. Despite these limitations, some advocate screening for hypercortisolaemia even in absence of symptoms or signs [24, 36]. Notably, in a prospective study which screened for Cushing’s syndrome in 68 patients with incidental pituitary adenomas, biochemical tests were positive in 7.3% with histological confirmation of Cushing’s disease in 4.4% of them [36]. Despite debate, most do not advocate screening for hypercortisolism in the evaluation of an incidentally detected pituitary microadenoma [22, 25, 26, 37], an opinion seemingly shared by majority of practicing UK clinicians particularly during the recent years.

Approximately 97%, 95% and 88% of 2021 survey respondents would screen for hypopituitarism, measuring LH, FSH and gonadal hormones, TSH and paired free T4 and morning cortisol, respectively. Interestingly, 6.7% would measure plasma and urine osmolalities aiming to investigate for diabetes insipidus. It is of note that dynamic testing for the hypothalamo-pituitary-adrenal axis would be performed by a higher number of clinicians in the 2009 survey (30.4% *vs* 11.4%), whereas morning cortisol would be used more frequently by the 2021 survey participants (87.9% *vs* 62.6%). At baseline, most clinicians share the opinion of screening for hypopituitarism, and request investigations as suggested by the 2016 Endocrine Society clinical practice guideline on hormonal replacement in adults with hypopituitarism [38]. The 2011 Endocrine Society guidelines on pituitary incidentalomas recommend screening for hypopituitarism for all microincidentalomas, particularly for tumours sized between 6 and 9 mm [22]. The 2021 German guideline on the diagnosis of clinically non-functioning pituitary adenomas similarly recommends screening for all anterior pituitary hormones at initial presentation, except for cortisol, in which measurement is recommended for larger tumours only (≥ 6 mm) [24]. Others stipulate the evaluation for any form of hypopituitarism is unnecessary for smaller tumours (< 5 mm), but for clinicians to consider assessment for those measuring > 5 mm [23, 26, 37, 39]. Debate most likely arises due to variance in the reported rates of hypopituitarism [12–16, 19, 40]. Furthermore, whilst some provide guidance based on size of microadenoma [23, 26, 37, 39], evidence to support a specific threshold is still lacking. Of note, two recent studies with a comparatively large cohort of clinically NFPAs were unable to demonstrate a significant difference in the prevalence of hypopituitarism between those smaller than 5 mm and those sized 5–9 mm [16, 18].

In the 2021 survey, a smaller rate of participants would organise visual assessment (15% *vs* 25%). Guidelines do not recommend formal visual assessment if the tumour is not abutting the optic pathway [22, 23, 25]. Nevertheless, one may choose to request visual field evaluation to use as a baseline in case of future tumour growth.

Respondents of 2021 survey were split on approach to further biochemical surveillance, with 47.2% opting for annual pituitary function assessment, compared to 52.1% who would re-test only in the presence of tumour growth or new, relevant clinical manifestations. Such divide in opinion is interesting as most guidelines recommend re-evaluation of pituitary function only in the presence of new relevant manifestations or new tumour growth [22, 23, 25, 26]. Indeed, in the meta-analysis performed by Fernández-Balsells et al*.,* the risk of new endocrine dysfunction was 4 per 100 patient-years [20]. Nonetheless, the German guidelines recommend annual biochemical screening for the first three years [24]. The cost-effectiveness of such approach remains to be elucidated.

Whilst nearly all clinicians from both surveys would go on to perform repeat imaging after the first MRI, the duration of radiological follow-up was highly variable; 2021 respondents were more likely to perform imaging surveillance up to 3 years (81.7% *vs* 44.3%) and 5 years (86.6% and 72.9%); whilst less likely to continue imaging beyond 5 years (7% *vs* 24%). Remarkable variability was also demonstrated in the responses and comments on the imaging intervals. The Endocrine Society recommends MRI after 1 year, and if stable, every 1–2 years for three years, and less frequently thereafter [22]. Recent guidelines published by the German Society for Endocrinology recommend MRI yearly for the first three years, and providing no change in tumour size, further imaging should be conducted in accord with individual assessment and discussion with the patient [24]. Extending the interval between the first and second scan, with repeat MRI performed 3 years after the initial imaging has also been proposed [12, 16], whilst others do not recommend any radiological surveillance for tumours less than 5 mm [23, 26]. Opinion amongst experts on optimal imaging intervals for microNFPAs is thus debated, a conflict seemingly reflected in our results. Optimal length of imaging surveillance is not clear, and this relates with the lack of long-term monitoring studies. In most reports to date, growth of microNFPAs occurs in a minority of patients; Karavitaki et al*.* [11] in a series of presumed microNFPAs followed-up for a mean period of 42 months reported 19% cumulative probability of tumour growth at 4 years. In a study of 271 patients with microNFPAs followed-up for a median period of 29 months, growth incidence was 2.1 per 100 person-years (95% CI 1.4–3.3) [16]. Notably, no significant difference in tumour growth rates between smaller and larger microNFPAs has been reported [16, 41]. Arguably of most significance, those that do grow rarely amount to any significant clinical consequence or require surgical intervention during the generally short follow-up periods [12, 20]. However, exceptions have been reported, with tumour enlargement occasionally managed by surgery [13, 18]. Given the slow growth potential of these lesions, the option of stopping imaging surveillance at 3 or at 5 years could be challenged necessitating longer follow-up studies to reliably inform on the safety of cessation of monitoring.

Tumour size, older age, and patient preference were the most common factors influencing patient discharge in the 2021 survey. Whilst endocrine guidelines do not provide specific recommendations on this, some advice against surveillance (biochemical or radiological) for microNFPAs sized < 5 mm, based on the low risk of tumour growth and hypopituitarism [23, 26, 37]. Given the available evidence suggesting that these tumours are slow-growing [10–12, 16], it is perhaps unsurprising that 69.8% of the respondents would have a lower threshold for discharging elderly patients, as the possibility of a grown microNFPA to cause clinically relevant problems and require surgical intervention is low in this group. In 22.3% of the clinicians, young age at tumour detection would deter them from discharge, presumably given the longer time frame for even slow-growing tumours to become clinically relevant. Patient preference would also influence decision to discharge, highlighting the importance of shared decision making and the need for clinicians to explore the patient’s management expectations and perceptions of their condition.

Comparing responses from clinicians working in secondary and tertiary care hospitals, choice of investigation and management was broadly similar, with exception of a greater number of secondary care clinicians choosing to rescan and discharge at one year (44.0% *vs* 23.1%), whilst tertiary care clinicians were more likely to perform annual assessment of pituitary function irrespective of tumour stability or absence of new manifestations (54% *vs* 36%). Observed differences may be influenced by resources available and/or individual clinician’s practice.

Our study allowed us to address the evolution of the UK practice on the topic with comprehensive questionnaires during a 13-year period and identify areas that still require clarification. The endorsement of both surveys by the SFE promoted national distribution and wide participation of the group of clinicians involved in the management of microNFPAs. Whilst pertinent to UK clinicians, we recognise our findings may be less applicable to non-UK centres, where clinician practice and health care systems may differ. Further national surveys would provide information on the approach in different health care systems. Inherent to all surveys, responses offered may not reflect practice implemented, and clinical approach may differ between responders and non-responders.

In conclusion, the clinical practice on the diagnosis and management of presumed microNFPAs has evolved in the UK. Currently, although a small rate of clinicians would consider imaging follow-up beyond 5 years, there is variability in the frequency of the imaging intervals and the number of MRIs chosen to be performed until discharge. Old age at tumour detection and lesion < 6 mm are main factors dictating decision to discharge. Yearly biochemical surveillance is performed by a considerable number of clinicians in the absence of tumour growth or clinical symptoms, despite international recommendations to the contrary. The uncertainty and lack of a unified approach to the management of microNFPAs highlighted by our study is shaped by the shortage of evidence exploring the long-term natural history of these tumours and necessitates generation of robust data on the risks of new hypopituitarism and clinically relevant tumour growth. Such risks must, in turn, be balanced against the potential impact of long-term surveillance on both patient and health care resources.

## Supplementary Information

Below is the link to the electronic supplementary material.

Supplementary file1 (PDF 48 kb)

Supplementary file2 (PDF 139 kb)

## Data Availability

Datasets are available upon request. **Ethical Approval.** Not applicable.
