# Supplementary material for: UK practice on incidentally detected non-functioning pituitary microadenomas: analysis of two national surveys during a 12-year interval
Source: Pituitary. 2022 Nov 25;26(1):94–104. doi: 10.1007/s11102-022-01290-4 (PMC9908737; doi:10.1007/s11102-022-01290-4)
Supplement: Supplementary file 1 — Supplementary file1 (PDF 48 kb) [file 11102_2022_1290_MOESM1_ESM.pdf]

Article title: **UK practice on incidentally detected non-functioning pituitary microadenomas: Analysis of two national surveys during a 12-year interval**

Journal name: **Pituitary**

Author names: **Ross Hamblin, Athanasios Fountas, Miles Levy, Niki Karavitaki**

Affiliation and e-mail address of the corresponding author: **Dr. Niki Karavitaki, MSc, PhD, FRCP**

Institute of Metabolism and Systems Research, College of Medical and Dental Sciences, University of Birmingham, IBR Tower, Level 2, Birmingham, B15 2TT, UK

E-mail: n.karavitaki@bham.ac.uk

Supplementary Figure 1: Case and questions of 2009 survey

CASE: A 25-year-old woman has a history of chronic non-specific headaches. Magnetic resonance imaging was performed and showed a 5 mm lesion in the pituitary gland consistent with a microadenoma. Except for headaches, the patient is asymptomatic. She has normal menses. Her physical examination is normal, and she has no galactorrhoea.

Which of the following tests would you order?

TEST	never →→	→→	→ always	
Prolactin	1	2	3	4
IGF-1	1	2	3	4
GH	1	2	3	4
LH/FSH	1	2	3	4
Oestradiol	1	2	3	4
Free T4	1	2	3	4
TSH	1	2	3	4
Serum cortisol (08.00-09.00 am)	1	2	3	4
Dynamic test of ACTH adrenal reserve	1	2	3	4
24hour urinary free cortisol	1	2	3	4
Low dose dexamethasone suppression test	1	2	3	4
Formal plotting of visual fields	1	2	3	4
Would you order a follow-up pituitary imaging procedure?<br>If yes, when______________________	1	2	3	4

Would you order other tests?<br>If yes, specify______________________	1 2 3 4

IGF-1 – Insulin-like Growth Factor 1, GH – Growth Hormone, LH – Luteinizing Hormone, FSH – Follicle Stimulating Hormone, TSH – Thyroid Stimulating Hormone, ACTH – Adrenocorticotropic Hormone
