# Supplementary material for: UK practice on incidentally detected non-functioning pituitary microadenomas: analysis of two national surveys during a 12-year interval
Source: Pituitary. 2022 Nov 25;26(1):94–104. doi: 10.1007/s11102-022-01290-4 (PMC9908737; doi:10.1007/s11102-022-01290-4)
Supplement: Supplementary file 2 — Supplementary file2 (PDF 139 kb) [file 11102_2022_1290_MOESM2_ESM.pdf]

Article title: **UK practice on incidentally detected non-functioning pituitary microadenomas: Analysis of two national surveys during a 12-year interval**

Journal name: **Pituitary**

Author names: **Ross Hamblin, Athanasios Fountas, Miles Levy, Niki Karavitaki**

Affiliation and e-mail address of the corresponding author: **Dr. Niki Karavitaki, MSc, PhD, FRCP**

Institute of Metabolism and Systems Research, College of Medical and Dental Sciences, University of Birmingham, IBR Tower, Level 2, Birmingham, B15 2TT, UK

E-mail: n.karavitaki@bham.ac.uk

Supplementary Figure 2: Questionnaire of 2021 survey

Incidentally detected non-functioning pituitary microadenomas:

A UK practice survey

1. Your position:
   A. Consultant Endocrinologist ☐
   B. Trainee in Endocrinology ☐
   C. Other (please provide details) ☐

   ____

2. Type of practice:
   A. Tertiary hospital ☐
   B. District General hospital ☐
   C. Private ☐
   D. Other ☐ (please provide details)

   ____

3. Requested investigations at first review after the incidental detection of a (presumed) pituitary **microadenoma** **(i.e. tumour with max diameter less than 1 cm)** (please tick all applicable options)
   A. Pituitary function tests ☐
   B. Visual assessment ☐
   C. Other ☐ (please add details)

____

4. Which pituitary function tests do you typically request? (please tick all applicable options)
   A. IGF-I ☐
   B. GH ☐
   C. FSH, LH and gonadal hormones ☐
   D. PRL ☐
   E. Morning cortisol ☐
   F. Short Synacthen test ☐
   G. TSH, fT4 ☐
   H. 24hr urine free cortisol ☐
   I. Overnight Dexamethasone suppression test ☐
   J. Plasma and urine osmolalities ☐
   K. Other ☐ (please add details)

   ____

5. If after the first review the investigations are consistent with a non-functioning pituitary **microadenoma** and providing you discovered no abnormalities in your selected investigations, do you:
   A. Discharge the patient? ☐ (please comment on what advice you would provide to the patient/GP)

   ____

   B. Continue monitoring? ☐

If you opted for A, no need to continue to further questions.

If you opted for B, please continue the survey.

6. What is your practice with imaging follow-up?
   A. Pituitary MRI at 1 year and if stable, discharge ☐
   B. Pituitary MRI at 1 and 2 years and if stable, discharge ☐
   C. Pituitary MRI at 1 ,2, and 3 years and if stable, discharge ☐
   D. Pituitary MRI at 1 and 2 years and then life-long clinical follow-up without discharge ☐

E. Other ☐ (please add details)

____

7. What is your practice with hormonal follow-up?

A. Yearly pituitary function tests until discharge ☐ (please define which)

____

B. I do not repeat pituitary function tests after initial assessment, unless clinical suspicion of new pituitary dysfunction or imaging has shown tumour enlargement ☐

C. Other ☐ (please add details)

____

8. What factors influence your decision to discharge the patient? (Please tick all that apply)

A. Young age at tumour detection ☐

B. Old age at tumour detection ☐

C. Maximum tumour diameter < 6 mm ☐

D. Patient Preference ☐

E. Other ☐ (please provide details)

____

9. Any other comments

____

____
